# Trends of HIV incidence and prevalence among men who have sex with men in Beijing, China: Nine consecutive cross-sectional surveys, 2008-2016

**DOI:** 10.1371/journal.pone.0201953

**Published:** 2018-08-09

**Authors:** Qiang Chen, Yanming Sun, Weidong Sun, Mingqiang Hao, Guiying Li, Xueli Su, Ruolei Xin, Hongyan Lu

**Affiliations:** Institute for AIDS/STD Control and Prevention, Beijing Center for Disease Control and Prevention, Beijing Center for Preventive Medical Research, Beijing, China; National and Kapodistrian University of Athens, GREECE

## Abstract

**Background:**

Sexual transmission of HIV among men who have sex with men (MSM) increased markedly in China during the past decade. HIV incidence is a critical indicator in HIV surveillance and we use a HIV-1 BED-capture-enzyme immunoassay (BED-CEIA) to examine the incidence among MSM in Beijing from 2008 to 2016. Risk factors related to recent HIV infection were also assessed.

**Methods:**

Consecutive cross-sectional surveys on MSM were conducted yearly from 2008 through 2016. Demographic and behaviors data were collected. HIV status was determined and HIV positive specimens were tested for recent infection using BED-CEIA. Specimens with ODn values≤0.8 were considered recently infected, HIV incidence rates and prevalence were then calculated. Risk factors associated with recent HIV infection were assessed by univariate and multivariable logistic regression.

**Results:**

From 2008 to 2016, the numbers of eligible participants in the nine consecutive years ranged from 472 to 616. All the 261 eligible HIV-positive specimens were subjected to recent HIV infection testing. HIV prevalence ranged from 5.0% (3.3%-6.8%) to 10.2% (7.8%-12.7%), and incidence ranged from 1.57% (0.19%-2.95%) to 6.63% (3.65%-9.61%). MSM who never or sometimes used condoms during anal sex with men in the past 6 months (aOR = 1.515, 95%CI: 1.016–2.257, p = 0.041), or having syphilis infection (aOR = 1.561, 95%CI: 0.946–2.575, p = 0.081) were more likely to be recently infected with HIV. Being a Beijing resident (aOR = 0.409, 95%CI: 0.212–0.790, p = 0.008), or having only one male anal sex partner in the past 6 months (aOR = 0.467, 95%CI: 0.220–0.994, p = 0.048) were associated with a lower risk for recent HIV infection.

**Conclusions:**

The HIV incidence fluctuated among MSM in Beijing. Unprotected anal sex, having multiple sex partners, being a non-registered Beijing resident and having a syphilis infection play important roles in the recent HIV infection. Effective intervention measures for HIV and syphilis control and prevention should be continuously strengthened.

## Introduction

By the end of 2014, there were 501,000 reported people living with HIV/AIDS in China. Sexual transmission is the primary mode of HIV transmission with sexual transmission among men who have sex with men (MSM) increasing markedly [1]. In 2011, a total of 48,000 individuals had newly diagnosed HIV infections in China; 29.4% of them were attributed to homosexual transmission [2]. According to case reports from 2006–2014 in China, the numbers of male homosexual transmissions shows uptrend. Of new cases diagnosed each year, the male homosexual transmission rate increased from 2.5% in 2006 to 25.8% in 2014[1]. In 2008–2009, a massive cross-sectional survey on MSM in 61cities in China was conducted and an overall prevalence of 4.9% for HIV was found [3].The HIV prevalence is even higher among MSM in some large Chinese cities: 9.5% in Harbin in 2011[4], 11.4% in Guangzhou in 2013[5], with an average of 9.9% in seven other big cities in 2013[6].

Beijing is the capital of China with a population of more than 20 million, and it is one of the world’s most populous cities. The city attracts people from all over the country. During the past few years in Beijing, the percentage of HIV infections transmitted by sexual contact increased from 87.1% in 2011 to 96.9% in 2016[7]. HIV has rapidly spread among the MSM population in the city in recent years, while the proportion of MSM among newly identified HIV cases increased from 61.7% in 2011 to 73.9% in 2016, significantly higher than that of heterosexual transmission (23%) [7].

HIV incidence is the rate of new infections in a population in a specified time period and it is a critical indicator in HIV surveillance. Estimations of HIV incidence are needed to identify the status and monitor the trends of the HIV epidemic, and to evaluate interventions for HIV prevention [8]. Cohort studies on HIV incidence estimates are complicated, laborious and time-consuming, making this research approach difficult to start and expensive to maintain. Meanwhile, loss to follow-up and selection bias may lead to unreliable incidence estimates [9].Alternatively, HIV incidence can be estimated by cross-sectional surveys through laboratory-based assays. During the past decade, several laboratory incidence assays have been conducted, and the HIV-1 BED-capture-enzyme immunoassay (BED-CEIA) being the most commonly used for HIV surveillance. The method is an IgG capture enzyme immunoassay used to detect and distinguish recent from long-term HIV-1 infections, and has been used widely to estimate HIV incidence in many areas, both in China [10–14] and outside of China [15–19]. While limited investigation was found to examine the trend of HIV incidence and factors causing the trend, especially in a metropolitan of huge mobile population for a long period.

In this report, we use BED-CEIA to examine HIV incidence among MSM in Beijing over a nine-year period (2008–2016), and to determine the current status of trends in incidence. Risk factors related to recent HIV infection were also investigated.

## Materials and methods

### Study design and participants

Consecutive cross-sectional surveys were conducted yearly from 2008 through 2016 in Beijing. The target population was MSM aged 18 years or over, who reported oral or anal sex with at least one male sex partner in the past year.

### Sampling and recruitment

For each year, 10 MSMs with different demographic characteristics were selected as the survey seeds and three recruitment cards were issued to each of them. They could then select three other MSMs from their friends to participate in the survey. The seeds should meet the following inclusion criteria: a) Be willing to recruit other MSM participants; b) Have a wide range of contacts in MSM community; c) The selection of seeds requires all types of demography. Each participant must have a recruitment card to participate in the survey. After the completion of his survey he will also get three recruitment cards. Recruitment continued until the number of participants reached around 600 for each year.

After written informed consent was obtained from the participants, each subject received an anonymous interview to collect information. The interviews were face-to-face and carried out by skilled interviewers in a private room at the voluntary counseling and testing (VCT) clinic of the Beijing Center for Disease Control and Prevention (Beijing CDC). A questionnaire included demographic information, HIV transmission related risk behaviors and antiretroviral therapy (ART) use. Mobile phone numbers or email addresses were also provided by the participants for laboratory testing results notification when necessary. A reward of 50 Chinese Yuan (CNY) cash (approximately 7–8 US dollars) was given for participation in the questionnaire survey. As a part of national HIV sentinel surveillance program, this study was approved by the Institutional Review Board of the National Center for AIDS/STD Control and Prevention, China CDC.

### HIV antibody and BED-CEIA testing

A volume of 3-5mL venous blood was collected from each participant. Plasma was separated from the blood for HIV testing. The screening test was performed by using two enzyme-linked immunoassays (ELISA; Diagnostic Kit for Antibody to HIV, BioMerieux, Boxtel, The Netherlands; Beijing Wantai Biological Pharmacy Enterprise Co.,Ltd, Beijing, China).All ELISA positive samples were confirmed by Western blot(WB; MP Biomedical Asia Pacific Pte. Ltd, Singapore) to determine HIV status.

HIV positive specimens were tested for recent infection using BED-CEIA except in the following conditions: (1) AIDS cases, (2) CD4+ T-cell counts<200, (3) the known long-term HIV infections or patients who receiving ART. The BED-CEIA was conducted according to the manufacturer’s instructions (Sedia BED-CEIA HIV-1 incidence EIA, Sedia Biosciences Corporation, Portland, OR, USA).Test specimens were initially run singly. The optical density (OD) values of test specimens were normalized by a ratio using a calibrator (specimen OD/calibrator OD) to minimize inter-run variations. Specimens with ODn≤1.2 were tested in triplicate to confirm their values. In confirmatory testing, specimens with ODn values≤0.8 were considered recent infections, while specimens with ODn>0.8 were classified as chronic infections.

### Calculation of incidence

HIV incidence is defined as the number of new HIV infections occurring in a population, usually expressed as a rate of infection per 100 persons per unit time [20]. The incidence rate was calculated using the McDougal formula described below [21]:

$$I=\frac{F\times (365/w)\times R}{N+F\times (365/w)\times R/2}\times 100\%,$$

where *F* is an adjustment factor; *w* is the mean window period of HIV recent infection detected by BED-CEIA (*w* = 168 days in China) [22, 23]. *R* is the number of patients who were classified as having recent HIV infections by BED-CEIA and *N* is the number of cases who tested HIV-negative. The adjustment factor was calculated as follows [21]:

$$F=\frac{(R/P)+\gamma -1}{\left(R/P)\times (\alpha -\beta +2\gamma -1\right)}\times 100\%,$$

where *P* is the total number of patients who tested HIV-positive, α is the sensitivity of the BED-CEIA, β is the specificity of BED-CEIA for the samples whose infected time between one week and two weeks, while γ is the specificity of BED-CEIA for the samples whose infected time beyond two weeks. Here in China, α is 0.8098, β is 0.7571 and γ is 0.9315.

The 95% confidence interval (CI) for the incidence estimate is given by the following formula:

$$95\%CI=\frac{\pm 1.96I}{\sqrt{R}}$$

### Statistical analysis

Questionnaire data were double entered and cleaned using EpiData software (version 3.1, Epidata Association, Odense, Denmark). STATA 14 was used to perform linear regression analyses and Wilcoxon test for trend, comparing differences of demographic and behavioral characteristics between years, as well as describing the trend over time. Univariate and multivariable logistic regression were performed using SPSS software (Version 18.0,SPSS, Inc.,Chicago, IL,USA) to assess risk factors associated with recent HIV infection. Univariate analyses were conducted first. Variables with a p-value less than 0.1 were included in the multivariable forward stepwise logistic regression. All statistical significance test results are reported as p-values; those differences with less than 0.05 were considered as a statistically significant.

## Results

From 2008 to 2016, the numbers of eligible participants in the nine consecutive years were 614, 616, 602, 579, 600, 600, 600, 472 and 600 respectively. For each year, participants were recruited during April to July. MSM participants’ demographic characteristics and certain sexual behaviors are shown in Table 1.During the nine-year period, increasing trends were found in the proportion of participants aged 40 or older (p = 0.011), being married/cohabiting (p = 0.011) or divorced/widowed (p = 0.026), monthly income over 4000 CNY (p = 0.007) and always using condom during anal sex with a man in the past 6 months (p = 0.048).Decreasing trends were found in the proportion of MSM aged 24 or younger (p = 0.014), being single(p = 0.011), monthly income below 4000 CNY (p = 0.007) and sometimes using condom during anal sex with a man in the past 6 months (p = 0.048). No trend was found for the variables of registered residence, nationality, education level, occupation or syphilis infection during the nine-year period. The syphilis prevalence fluctuated, with the highest prevalence in 2012(18.5%) and the lowest in 2013(10.5%).

**Table 1:** Demographic characteristics and main behaviors of MSM in sentinel surveillance surveys in Beijing, 2008–2016.

Variable	2008 (N = 614) n (%)	2009 (N = 616) n (%)	2010 (N = 602) n (%)	2011 (N = 579) n (%)	2012 (N = 600) n (%)	2013 (N = 600) n (%)	2014 (N = 600) n (%)	2015 (N = 472) n (%)	2016 (N = 600) n (%)	Trend Test	
										z-value	p-value
**Age**											
** ≤24**	195 (31.8)	182 (29.6)	192 (31.9)	178 (30.7)	146 (24.3)	100 (16.7)	83 (13.8)	80 (17.0)	75 (12.5)	-2.45	0.014
** 25–39**	358 (58.3)	355 (57.6)	357 (59.3)	334 (57.7)	374 (62.4)	374 (62.3)	398 (66.3)	281 (59.5)	349 (58.2)	1.16	0.246
** ≥40**	61 (9.9)	79 (12.8)	53 (8.8)	67 (11.6)	80 (13.3)	126 (21.0)	119 (19.9)	111 (23.5)	176 (29.3)	2.55	0.011
**Marital status**											
** Single**	491 (80.0)	478 (77.6)	495 (82.2)	457 (78.9)	456 (76.0)	420 (70.0)	401 (66.8)	325 (68.8)	377 (62.8)	-2.55	0.011
** Married or cohabiting**	89 (14.5)	99 (16.1)	85 (14.1)	84 (14.5)	107 (17.8)	127 (21.2)	147 (24.5)	116 (24.6)	155 (25.8)	2.53	0.011
** Divorced or widowed**	34 (5.5)	39 (6.3)	22 (3.7)	38 (6.6)	37 (6.2)	53 (8.8)	52 (8.7)	31 (6.6)	68 (11.4)	2.22	0.026
**Registered residence**											
** Beijing**	151 (24.6)	129 (20.9)	131 (21.8)	108 (18.7)	115 (19.2)	141 (23.5)	122 (20.3)	105 (22.2)	111 (18.5)	-1.13	0.258
** Outside Beijing**	463 (75.4)	487 (79.1)	471 (78.2)	471 (81.3)	485 (80.8)	459 (76.5)	478 (79.7)	367 (77.8)	489 (81.5)	1.13	0.258
**Nationality**											
** Han**	578(94.1)	584 (94.8)	559 (92.9)	543 (93.8)	574 (95.7)	551 (91.8)	557 (92.8)	438 (92.8)	567 (94.5)	-0.92	0.356
** Other**	36(5.9)	32 (5.2)	43 (7.1)	36 (6.2)	26 (4.3)	49 (8.2)	43 (7.2)	34 (7.2)	33 (5.5)	0.92	0.356
**Education level**											
** High school or lower**	303 (49.3)	313 (50.8)	253 (42.0)	316 (54.6)	331 (55.2)	281 (46.8)	296 (49.3)	218 (46.2)	336(56.0)	0.43	0.670
** College or higher**	311 (50.7)	303 (49.2)	349 (58.0)	263 (45.4)	269 (44.8)	319 (53.2)	304 (50.7)	254 (53.8)	264 (44.0)	-0.43	0.670
**Occupation**											
** Employed**	585(95.3)	553 (89.8)	546 (90.7)	528 (91.2)	575 (95.8)	542 (90.3)	564 (94.0)	444 (94.1)	568 (94.7)	0.61	0.540
** Not employed**	29(4.7)	63 (10.2)	56 (9.3)	51 (8.8)	25 (4.2)	58 (9.7)	36 (6.0)	28 (5.9)	32 (5.3)	-0.61	0.540
**Monthly income(CNY)**											
** <4000**	468(76.2)	496 (80.5)	415 (68.9)	402 (69.4)	393 (65.5)	337 (56.2)	300 (50.0)	189 (40.0)	260 (43.3)	-2.69	0.007
** ≥4000**	146(23.8)	120 (19.5)	187 (31.1)	177 (30.6)	207 (34.5)	263 (43.8)	300 (50.0)	283 (60.0)	340 (56.7)	2.69	0.007
**Number of male anal sex partners in the past 6 months**^**a**^											
** 1**	189(34.4)	198(34.9)	213(37.4)	196(36.2)	203(36.3)	181(32.9)	169(31.6)	167(38.7)	226(43.5)	0.99	0.322
** 2**	154(28.1)	136(23.9)	118(20.7)	132(24.4)	111(19.8)	141(25.6)	125(23.4)	114(26.4)	113(21.8)	-0.47	0.637
** 3–9**	157(28.6)	178(31.3)	188(33.1)	156(28.8)	187(33.4)	174(31.7)	187(35.0)	120(27.8)	143(27.6)	-0.52	0.604
** ≥10**	49(8.9)	56(9.9)	50(8.8)	57(10.6)	59(10.5)	54(9.8)	54(10.0)	31(7.1)	37(7.1)	-0.92	0.356
**Condom use during anal sex with a man in the past 6 months**^**a**^											
** Never or sometimes**	270(49.2)	315(55.5)	307(54.0)	268(49.5)	220(39.3)	266(48.4)	206(38.5)	176(40.7)	223(43.0)	-1.98	0.048
** Always**	279(50.8)	253(44.5)	262(46.0)	273(50.5)	340(60.7)	284(51.6)	329(61.5)	256(59.3)	296(57.0)	1.98	0.048
**Syphilis infection**	75 (12.2)	75 (12.2)	78 (13.0)	72 (12.4)	111 (18.5)	63 (10.5)	75 (12.5)	62 (13.1)	92 (15.3)	1.44	0.149

MSM, men who have sex with men; CNY, Chinese Yuan

^a^ Not including MSM who had no anal sex in the past 6 months

**Fig 1:**
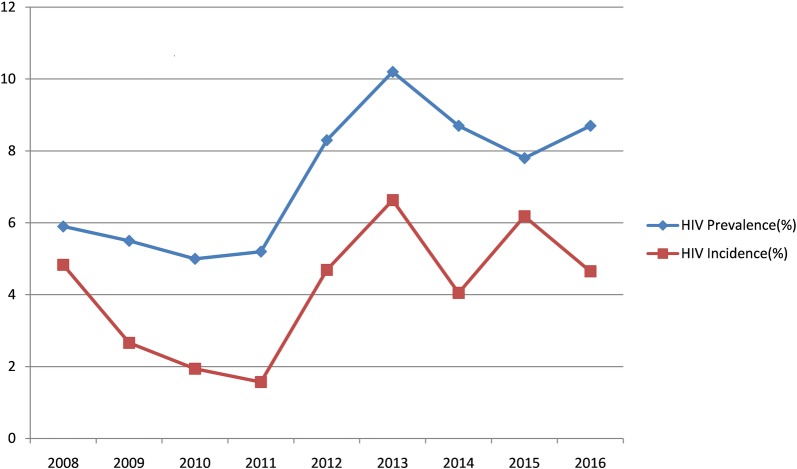
Trends of HIV incidence and prevalence among MSM in Beijing, 2008–20016.

As shown in Table 2 and Fig 1, the HIV prevalence among MSM remained at a relatively low level from 5.9% in 2008 to 5.2% in 2011, while it increased significantly to 10.2% in 2013, and then dropped slightly but kept a relatively high level between 2013 and 2016, reaching 8.7% in 2016. A total of 121 HIV-positive participants from the nine-year survey period were determined as previously reported HIV cases or the known long-term HIV infections, their specimens were excluded from testing of recent infection with BED-CEIA. From 2008 to 2016, all the 261 HIV-positive specimens that should be detected with BED-CEIA were available and subjected to recent HIV infection testing. The estimated HIV incidence among MSM showed a substantial decrease from 4.83% (95% CI:2.30%-7.36%) in 2008 to 1.57% (95% CI:0.19%-2.95%) in 2011,while a sharp increase to 6.63% (95% CI:3.65%-9.61%) in 2013 was observed, the incidence then fluctuated at a relatively high level during the period of 2013–2016, with rates of 6.63% (95% CI:3.65%-9.61%), 4.05% (95% CI:1.76%-6.34%), 6.18% (95% CI:2.94%- 9.42%) and 4.65% (95% CI:2.21%-7.08%), respectively.

**Table 2:** HIV Prevalence and estimated incidence of MSM in sentinel surveillance surveys in Beijing, 2008–2016.

Year	Participants, No.	HIV-positive cases, No.	HIV prevalence, %	HIV-negative cases (N), No.	Previously reported HIV cases, No.	Participants except reported HIV case, No.	Cases tested with BED (P), No.	Recent HIV infections (R), No.	Estimated HIV incidence (I), % (95% CI)
2008	614	36	5.9(4.2–7.8)	578	12	602	24	14	4.83(2.30–7.36)
2009	616	34	5.5(3.7–7.5)	582	15	601	19	8	2.66(0.82–4.50)
2010	602	30	5.0(3.3–6.8)	572	12	590	18	6	1.94(0.39–3.49)
2011	579	30	5.2(3.5–7.1)	549	11	568	19	5	1.57(0.19–2.95)
2012	600	50	8.3(6.2–10.7)	550	12	588	38	14	4.69(2.23–7.14)
2013	600	61	10.2(7.8–12.7)	539	19	581	42	19	6.63(3.65–9.61)
2014	600	52	8.7(6.7–11.0)	548	19	581	33	12	4.05(1.76–6.34)
2015	472	37	7.8(5.5–10.0)	435	9	463	28	14	6.18(2.94–9.42)
2016	600	52	8.7(6.3–11.0)	548	12	588	40	14	4.65(2.21–7.08)

HIV, human immunodeficiency virus; MSM, men who have sex with men; CI, confidence interval; N, the number of cases who were tested HIV-negative; P, the number of patients who tested with BED, here it does not include previously reported HIV cases; R, the number of patients who were classified as recent HIV infection by BED; I, estimated HIV incidence.

Factors associated with recent HIV infection are shown in Table 3, with all the HIV negative cases as controls during the survey period (2008–2016). In the univariate analysis, significant differences between MSM who recently infected with HIV and those who were HIV negative were found by registered residence, education level, number of male anal sex partners in the past 6 months, condom use during anal sex with a man in the past 6 months, and syphilis infection status; while no significant difference was found in participants’ age, marital status, nationality, occupation or monthly income. In the multivariable analyses, MSM who never or sometimes using condom during anal sex with a man in the past 6 months (aOR = 1.515, 95%CI: 1.016–2.257, p = 0.041, compared with those always using condom), having a syphilis infection (aOR = 1.561, 95%CI: 0.946–2.575, p = 0.081) were more likely to be recently infected with HIV; while being a Beijing resident (aOR = 0.409, 95%CI: 0.212–0.790, p = 0.008, compared with those not being a Beijing resident), having only one male anal sex partner in the past 6 months(aOR = 0.467, 95%CI: 0.220–0.994, p = 0.048, compared with having more than one partners) were associated with lower risk for recent HIV infection.

**Table 3:** Risk factors associated with recent HIV Infection among MSM in sentinel surveillance surveys in Beijing, 2008–2016, by logistic regression model.

Variable	HIV-negative cases		Recent HIV infections		OR (95% CI)	p-Value	aOR (95% CI)	p-Value
	n	%	n	%				
**Age**								
** ≤24**	1151	23.5	24	22.6	1.054(0.557–1.997)	0.871		
** 25–39**	2941	60.0	66	62.3	1.135(0.654–1.970)	0.653		
** ≥40**	809	16.5	16	15.1	1			
**Marital status**								
** Single**	3638	74.2	68	64.1	0.708(0.350–1.432)	0.337		
** Married or cohabiting**	922	18.8	29	27.4	1.192(0.558–2.543)	0.650		
** Divorced or widowed**	341	7.0	9	8.5	1			
**Registered residence**								
** Beijing**	1076	22.0	11	10.4	0.412(0.220–0.771)	0.006	0.409(0.212–0.790)	0.008
** Outside Beijing**	3825	78.0	95	89.6	1		1	
**Nationality**								
** Han**	4602	93.9	97	91.5	0.700(0.350–1.400)	0.314		
** Other**	299	6.1	9	8.5	1			
**Education level**								
** High school or lower**	2389	48.7	62	58.5	1.482(1.003–2.189)	0.048		
** College or higher**	2512	51.3	44	41.5	1			
**Occupation**								
** Employed**	4566	93.2	99	93.4	1.038(0.478–2.251)	0.926		
** Not employed**	335	6.8	7	6.6	1			
**Monthly income(CNY)**								
** <4000**	2990	61.0	64	60.4	0.974(0.657–1.443)	0.895		
** ≥4000**	1911	39.0	42	39.6	1			
**Number of male anal sex partners in the past 6 months**^**a**^								
** 1**	1652	37.0	19	18.6	0.413(0.195–0.875)	0.021	0.467(0.220–0.994)	0.048
** 2**	1064	23.8	27	26.5	0.911(0.448–1.854)	0.798	1.027(0.502–2.099)	0.943
** 3–9**	1359	30.4	45	44.1	1.189(0.609–2.321)	0.612	1.269(0.649–2.482)	0.486
** ≥10**	395	8.8	11	10.8	1		1	
**Condom use during anal sex with a man in the past 6 months**^**a**^								
** Never or Sometimes**	2060	46.1	58	56.9	1.542(1.038–2.292)	0.032	1.515(1.016–2.257)	0.041
** Always**	2410	53.9	44	43.1	1		1	
**Syphilis infection**								
** Yes**	615	12.5	21	19.8	1.722(1.060–2.797)	0.028	1.561(0.946–2.575)	0.081
** No**	4286	87.5	85	80.2	1		1	

OR, odds ratio; aOR, adjusted odds ratio; CI, confidence interval; CNY, Chinese Yuan; HIV, human immunodeficiency virus; MSM, men who have sex with men.

^**a**^ Not including MSM who have no anal sex in the past 6 months

## Discussion

During the past decade, the main transmission route of HIV in China has shifted from injection drug use and unsafe plasma collection to sexual transmission [24]. There has been a rise in HIV infection among MSM in China in recent years and most of these cases are localized in Chinese large cities [25]. In Beijing, the HIV epidemiology is characterized by the relatively high prevalence of HIV infection among MSM and a transmission route dominated by male homosexual behavior. Our study is the first to examine the trend of HIV incidence by using laboratory tests in Beijing. Multi-year surveys on HIV incidence estimation conducted by laboratory-based method are also limited in China.

Our study found an increasing HIV prevalence among MSM in Beijing during a period, coinciding with the increase in other Chinese large cities [4, 5, 25, 26].Simultaneously, we also found a trend in the increasing proportion of participants who always use condom during anal sex with a man in the past 6 months, addressing the concern as to the protective effect of the condom use. In our study, condom use in anal sex was self-reported by participants themselves. Thus some receptive subjects may not be certain about the condom use of their insertive partners, or may be deceived by their insertive partners about the use of condoms during receptive anal intercourse. Thus, the reported rise in condom use may result from recall bias and reporting bias. On the other hand, the rising HIV prevalence among MSM means that subjects have more chance for personal contact with infected MSM, while the low value of elevated condom use was not high enough to offset the increased risk of infection.

Consistent with findings from other surveys [13, 18], risk factors associated with recent HIV infections were identified in this study. Condom use promotion and related health education are two of the most important behavioral interventions among high-risk population such as MSM in Beijing. In our study, the increasing trend of the proportion of participants who always use condoms during anal sex with a man in the past 6 months, with no decreasing HIV incidence trend, suggests that the effectiveness of condom use may counteracted by increased risk of infection, and that condom use promotion alone is not effective to prevent HIV transmission among high-risk population. Other intervention strategies should be strengthened, such as expanding HIV testing, early identification of HIV infection and timely treatment. Syphilis infection was also associated with increased risk of HIV acquisition and transmission in our study. The prevalence of syphilis infection remained relatively high level (around 12.0%) during the study period, consistent with the result of a large investigation in China [3]. Syphilis can facilitate HIV transmission among MSM, because syphilis lesions increase the sexual transmission efficiency of the HIV virus [27,28]. Syphilis played a role in the spread in HIV among MSM in our study and its prevalence was higher than that of HIV. These findings suggest that we reinforce our efforts in behavioral interventions focus on preventing syphilis. Theoretically, such measures should also reduce the chance of HIV transmission.

Our study identified being a Beijing registered resident and having a single male anal sex partner in the past 6 months as protective factors for recent HIV infection. Beijing has a large population of over 20 million, nearly half of the citizens are not Beijing registered residents and most of them belong to mobile populations. From 2008 to 2016, MSM of non-registered Beijing residents accounted for about 80% of all participants in our survey each year. Studies have shown that increased mobility is associated with increased levels of “recent” sexual behavior and increased risk of HIV infection [29, 30]. Subjects who have a single sex partner are less susceptible to infection with HIV than those who have multiple sex partners. The unstable lifestyle and the lack of family communications in mobile populations, may lead to more sexual partners and more unsafe sexual behaviors. Therefore, mobile populations become the main target of HIV prevention in Beijing.

Our investigation is subject to several limitations. First, each year we had a limited number of subjects in the subsets of recent infections, so the findings may not be generalizable to all MSM in a city of very large population. Second, concerns about using the BED-CEIA assay for estimation of HIV incidence have been raised due to the misclassification of long-term infections as recently acquired, such misclassification results in overestimating HIV incidence. To reduce our potential distortions during the incidence estimates, long-term infections were removed from BED-CEIA testing and a Chinese local adjustment factor was utilized in the incidence calculation [23]. Despite trends in HIV incidence is our first concern, a new generation of laboratory methods for estimating HIV-1incidence such as limiting antigen avidity enzyme immunoassay (Lag Avidity assay) will also be applied in further investigations. Finally, since the behavioral information was self-reported, some high-risk behaviors may have been underreported and reporting bias as well as recall bias may exist, leading to a potential underestimation of the role of risk factors. To minimize the biases, training courses were provided annually to the interviewers who administrated questionnaire.

In summary, our study illustrates an expanding HIV epidemic and an overall fluctuation in HIV incidence among MSM in Beijing, during 2008–2016. Unprotected anal sex, having multiple sex partners, being a non-registered Beijing resident and having syphilis infection may play important roles in the recent HIV infections. Effective intervention measures for HIV and syphilis prevention and control should be continuously strengthened in the MSM population especially among those who are migrants. Expanding HIV /syphilis testing, condom use promotions, early identification of HIV/syphilis infection and timely treatment should be major foci of public health efforts to reduce HIV infections in China MSM population.
